# Transcriptional regulation of the GTP cyclohydrolase I gene via the NF-κB pathway by bacterial and viral immune stimulants

**DOI:** 10.1093/jb/mvaf060

**Published:** 2025-10-23

**Authors:** Miori Ozawa, Satoshi Hara, Masaru Sakamoto, Takahiro Suzuki, Shuhei Niiyama, Yasuyuki Kakihana, Hiroshi Ichinose

**Affiliations:** School of Life Science and Technology, Institute of Science Tokyo, 4259-B7, Nagatsuta-cho, Midori-ku, Yokohama 226-8501, Japan; School of Life Science and Technology, Institute of Science Tokyo, 4259-B7, Nagatsuta-cho, Midori-ku, Yokohama 226-8501, Japan; Department of Emergency and Intensive Care Medicine, Kagoshima University Graduate School of Medical and Dental Sciences, Kagoshima University, 37-1 Kamiarata-cho, Kagoshima 890-8760, Japan; School of Life Science and Technology, Institute of Science Tokyo, 4259-B7, Nagatsuta-cho, Midori-ku, Yokohama 226-8501, Japan; School of Life Science and Technology, Institute of Science Tokyo, 4259-B7, Nagatsuta-cho, Midori-ku, Yokohama 226-8501, Japan; Department of Biochemistry, School of Dentistry, Aichi Gakuin University, 1-100 Kusumoto-cho, Chikusa-ku, Nagoya 464-8650, Japan; Department of Emergency and Intensive Care Medicine, Kagoshima University Graduate School of Medical and Dental Sciences, Kagoshima University, 37-1 Kamiarata-cho, Kagoshima 890-8760, Japan; Department of Emergency and Intensive Care Medicine, Kagoshima University Graduate School of Medical and Dental Sciences, Kagoshima University, 37-1 Kamiarata-cho, Kagoshima 890-8760, Japan; School of Life Science and Technology, Institute of Science Tokyo, 4259-B7, Nagatsuta-cho, Midori-ku, Yokohama 226-8501, Japan

**Keywords:** GTP cyclohydrolase I, Macrophage, NF-κB, Tetrahydrobiopterin, Toll-like receptor ligands

## Abstract

Tetrahydrobiopterin (BH4) is an essential cofactor for biosynthesis of monoamines and nitric oxide. An excess of BH4 in infiltrated macrophages was reported to cause pain, while a certain level of BH4 is essential for cell survival and proliferation. GTP cyclohydrolase I (GCH) is a rate-limiting enzyme for the *de novo* synthesis of BH4. Our previous study showed that GCH expression was elevated by an enhancer region containing the C/EBP and Ets binding motifs in macrophage-like RAW264.7 cells when stimulated with lipopolysaccharide (LPS). In this study, we showed that poly(I:C) and R848, Toll-like receptors ligands for RNA viruses, increased GCH expression and BH4 levels in RAW264.7 cells as well as bacterial LPS. We examined the intracellular signaling pathway for the induction of the *Gch* gene, and found that inhibitors for the NF-κB pathway suppressed the GCH expression by these stimuli. We for the first time identified the region required for LPS-induced GCH expression to be the 5′-untranslted region of exon 1 consisting of 149 bp using a reporter experiment. We also demonstrated that the expression of GCH with LPS was strongly suppressed by an inhibitor of NF-κB in mouse intraperitoneal macrophages *in vivo*.

## Graphical Abstract

**Graphical Abstract:**
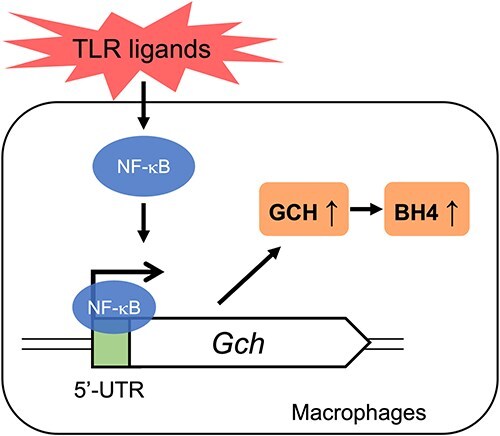

## Abbreviations

BH4: tetrahydrobiopterin
BH2: dihydrobiopterin
BP: biopterin
DHFR: dihydrofolate reductase
GCH: GTP cyclohydrolase I
LPS: lipopolysaccharide
NO: nitric oxide
NOS: nitric oxide synthase
NP: neopterin
poly(I:C): polyinosinic-polycytidylic acid
QDPR: *quinonoid* dihydropteridine reductase
TLCK: N^α^-tosyl-L-lysine chloromethyl ketone hydrochloride
TLR: Toll-like receptor
TH: tyrosine hydroxylase

Tetrahydrobiopterin (BH4) is an essential cofactor for aromatic amino acid hydroxylases (phenylalanine hydroxylase, tyrosine hydroxylase and tryptophan hydroxylase), nitric oxide synthase (NOS) and alkylglycerol monooxygenase *(**1**)*. In addition to the well-known functions, recent studies have highlighted its roles in T-cell proliferation and protection from ferroptosis *(**2**,*  *3**)*. Deficiency of BH4 is associated with phenylketonuria, neural disorders and endothelial dysfunction due to impaired phenylalanine and monoamine metabolisms, and dysregulation of the NOS activity.

BH4 is synthesized by three enzymatic reactions from GTP. GTP cyclohydrolase I (GCH), synthesizing dihydroneopterin triphosphate from GTP, is an initial and rate-limiting enzyme in the *de novo* synthesis of BH4. Therefore, the GCH activity is primary determinant of the BH4 levels, whereas regeneration of BH4 by *quinonoid* dihydropteridine reductase (QDPR) and dihydrofolate reductase (DHFR) in the regeneration pathway has some contribution to the intracellular BH4 levels *(**4**,*  *5**)* (see Fig. S1). An autosomal dominant defect in the GCH gene causes dopa-responsive dystonia (DYT5) *(**6**)*, and a mouse model of hyperphenylalaninemia, *hph-1*, is reported to be caused by decreased expression of the *Gch* gene *(**7**)*, indicating the significance of the GCH activity in the BH4 metabolism.

Dihydroneopterin triphosphate, which is the product of the GCH reaction, can be converted to neopterin (NP) through dephosphorylation and oxidation *(**8**)*. NP is synthesized in and excreted from monocytes and macrophages by immune stimulants such as lipopolysaccharide (LPS) and interferon-γ. NP is considered to be a side product of BH4-biosynthesis, because its physiological role is still remained unclear. However, NP is reported to be a good biomarker for immune activation in human *(**9**)*. NP levels in body fluids elevate by the inflammatory signals caused by bacterial and viral infection, and it has been suggested that the increases are more sensitive in viral infection than bacterial one *(**10**–**12**)*. Despite the physiological roles and metabolism of NP are not fully elucidated, the control mechanism for the GCH expression in immune cells is of value to reveal the intracellular signals of natural immunity induced by viral and bacterial infection.

Since BH4 has diverse physiological role, there are multiple mechanisms to regulate GCH expression depending on tissues and cell types. In monoaminergic cell lines, GCH expression was regulated by cAMP in SK-N-BE(2)M17 cells *(**13**)* and nerve growth factor (NGF) via Ras/MEK pathway in PC12D cells *(**14**)*. Upon immune activation, a cyclin-dependent kinase (CDK) inhibitor decreased NF-κB activation and expression levels of GCH and inducible NOS (iNOS) in macrophage-like RAW264.7 cells *(**15**)*. Moreover, GCH induction by interferon-γ and TNF-α in endothelial cells was reported to require JAK/STAT and NF-κB pathways *(**16**)*. However, transcriptional regulatory mechanisms for induction of GCH by various pathogens are largely unknown.

We previously reported that the induction of *Gch* by LPS required an enhancer region located in the intron, with binding of Ets and C/EBP to the enhancer region *(**17**)*. In this study, we examined polyinosinic-polycytidylic acid (poly(I:C)) and R848, which are analogues of Toll-like receptor (TLR) ligands for RNA viruses, induced *Gch* expression in RAW264.7 cells. We further addressed downstream signal of TLRs and showed that NF-κB pathway plays a crucial role in regulating Gch expression. Finally, we for the first time found that 5′-untranlated region (5’-UTR) is critical for the *Gch* induction, and that an NF-κB inhibitor dramatically suppressed LPS-induced GCH expression in mouse peritoneal macrophages by an *in vivo* experiment.

## Materials and Methods

### Cell culture and treatment

The mouse macrophage cell line RAW264.7 was cultured in Dulbecco’s modified Eagle medium with high glucose (Fujifilm-Wako, Osaka, Japan) containing 10% fetal bovine serum (Biowest, Nuaillé, France) and 1% penicillin/streptomycin (Thermo Fisher Scientific, Waltham, MA) at 37 °C under 5% CO_2_ atmosphere. The following TLR ligands were added to the medium; LPS from *Eschericia coli* O55:B5 (L2637, Sigma-Aldrich, St. Louis, MO), poly(I:C) (P1530, Sigma-Aldrich, St. Louis, MO), or R848 (resiquimod, SML0196, Sigma Aldrich, St. Louis, MO), dissolved in dimethyl sulfoxide. Inhibitors for NF-κB pathways, N^α^-tosyl-L-lysine chloromethyl ketone hydrochloride (TLCK) (200–20,141, Fujifilm, Tokyo, Japan) or JSH-23 (15,036, Cayman Chemical Co.ltd., MI); for MEK1/2 pathway, U0126 (V112A, Promega, Madison, WI); for p38 MAPK pathway, SB203580 (559,389, Merck, Darmstadt, Germany) were added to the cells 30 min before TLR ligands.

### Measurement of BH4 levels

BH4 levels were measured as oxidized biopterin (BP) using ultra-high performance liquid chromatography with a fluorescence detection (Shimadzu, Kyoto, Japan). Collected cell pellets were resuspended with 20 μL phosphate-buffered saline (PBS), and 100 μL of 0.4 M perchloric acid containing 0.1 mM ethylenediaminetetraacetic acid (EDTA) was added and homogenized by pipetting. After 30 min incubation on ice, the homogenates were centrifuged at 20,000 × *g* for 10 min at 4 °C. The precipitates were dissolved in 0.1 M NaOH, and their protein concentrations were determined with a DC protein assay kit (Bio-Rad, Hercules, CA). The supernatants were oxidized with the iodine solution consisting of 1% I_2_ and 2% KI under acidic condition. After the addition of 2% ascorbic acid to reduce the excess iodine, the solution was centrifuged at 20,000 × *g* for 10 min at 4 °C, and the supernatants were used for the analysis. BP was separated using a Shim-pack Velox Biphenyl column (Shimadzu, Kyoto, Japan) with 10 mM Na-phosphate (pH 3.5), and quantitated by fluorescence (excitation: 365 nm, emission: 475 nm).

### Measurement of GCH activities

Collected cells were lysed by freeze and thaw in 100 mM Tris–HCl (pH 8.0), 300 mM KCl, 2.5 mM EDTA and 10% Glycerol, and centrifugated. The supernatants were then collected and assayed as previously described *(**18**)*.

### Quantitative reverse transcription PCR (qRT-PCR)

Total RNA was isolated from the cells using RNeasy Mini Kit (QIAGEN, Hilden, Germany). cDNA was synthesized with random hexamer and SuperScript II Reverse Transcriptase (Invitrogen, California, USA). Quantitative PCR was performed using Thunderbird SYBR qPCR Mix (TOYOBO, Osaka, Japan). The Primers used were *Gch* forward, 5’-AGCAAGTCCTTGGTCTCAGTAAAC-3′ and reverse, 5’-ACCGCAATCTGTTTGGTGAGGC-3′; *Gapdh* forward, 5’-AGGTCGGTGTGAACGGATTTG-3′ and reverse, 5’-GGGGTCGTTGATGGCAACA-3′. The thermal profile consisted of initial denaturation at 95 °C for 30 sec followed by 45 cycles of 95 °C for 5 sec, 55 °C for 5 sec, and 72 °C for 20 sec with a final dissociation step. Expressions of mRNA were normalized to *Gapdh*.

### Western blot

Cells were collected and homogenized with RIPA buffer (50 mM Tris–HCl (pH 8.0), 150 mM NaCl, 0.5% Triton X-100, 0.1% SDS, 1 mM phenylmethylsulfonyl fluoride, 1 μg/ml leupeptin and 1 μg/ml pepstatin). The amounts of protein in the lysates were quantified with DC protein assay, and 20 μg protein of the lysate was loaded onto an SDS-PAGE gel. After electrophoresis and transfer to PVDF membrane, the amounts of GCH protein were detected by incubating with polyclonal GCH antibody (αGCH-N2, self-made) *(**19**)* at 4 °C overnight. The blots were incubated with secondary antibody (HRP-linked goat anti mouse/rabbit IgG; GE healthcare, Chicago, IL) for 1 h at room temperature and then washed. Chemi-luminescence generated from Immobilon Western (Merck) was detected with Ez-Capture MG/ST (ATTO, Tokyo, Japan).

### DNA transfection and luciferase assay

Cells were cultured in 24 well-plates in 500 μL medium on a day prior to transfection. The firefly luciferase reporter genes containing human GCH promoter region including 5’-UTR and enhancer region of various sizes were constructed as previously described *(**17**)*. A sea pansy luciferase vector, pRL-TK (Toyoink, Tokyo, Japan), was used as an internal control to normalize for variations in transfection efficiency. Firefly reporter plasmid (0.4 μg) and pRL-TK plasmid (0.08 μg) were diluted in 50 μL Opti-MEM reduced serum medium (Thermo Fisher Scientific), and 1.5 μL X-tremeGene HP DNA transfection reagent (Roche, Basel, Switzerland) was added and incubated at room temperature for 15 min. The DNA complex (50 μL) was added to each well, and the cultures were allowed to grow for 24 h, followed by LPS treatment for 24 h. Cells were assayed for firefly and sea pansy luciferase activity with a dual-luciferase reporter assay system (Promega).

### Animals and treatment

Wild-type male 9-week-old C57BL/6 J mice (Slc, Tokyo, Japan) were housed at 25 °C under a 12-h light/dark cycle and given free access to water and food. All animal experiments were performed in accordance with the general guidelines of Institute of Science Tokyo(D2022013). To induce peritoneal macrophages, 1 mL of sterilized 3.8% brewer’s thioglycolate medium was injected intraperitoneally to mice. After four days, mice were injected LPS (5 mg/kg) with or without BAY11–7082 (10 mg/kg; Tokyo Chemical Industry, Tokyo, Japan) dissolved in PBS/10% DMSO intraperitoneally. After 6 h of the injection, mice were sacrificed and peritoneal macrophages were harvested by intraperitoneal lavage.

### Bioinformatic analyses

The JASPAR (http://jaspar.genereg.net/) was used to predict the NF-κB binding site of the GCH promoter (pro5.2 k) and enhancer (Int332) sequences. The binding regions of NF-κB (NFKB1) on the GCH promoter and enhancer were analyzed using ChIP-Atlas *(**20**)*. Species we used were human (hg38) and mouse (mm10).

### Statistical analyses

All statistical analyses were performed with EZR software *(**21**)*. Student’s *t*-test or one-way ANOVA with Tukey’s and Dunnett’s comparison tests were used as indicated.

## Results

### The immune stimulation by TLR ligands enhanced intracellular BH4 levels

**Fig. 1:**
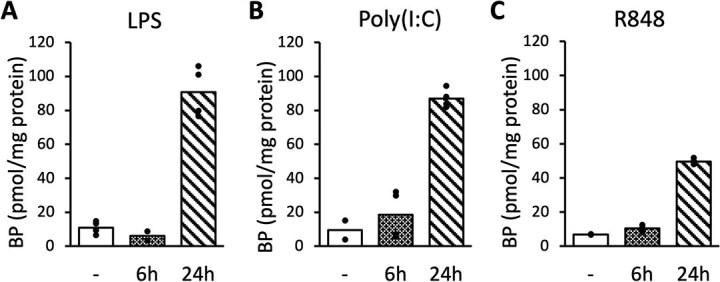
**Multiple TLR ligands upregulates intracellular BH4 level. A**, **B**, **C**. BP levels in RAW264.7 cells were measured 6 or 24 h after stimulation with 0.1 μg/ml LPS (**A**), 10 μg/ml poly(I:C) (**B**), and 0.1 μg/ml R848 (**C**). Control samples of LPS, poly(I:C) and R848 were treated with corresponding solvents for 24, 6 and 6 h, respectively. *n* = 2 for LPS 6 h, poly(I:C) control, and R848 control samples. *n* = 4 for other samples. Data are shown as means.

In our previous study, it was shown that stimulation with LPS, a ligand for TLR4 mainly recognizing bacterial antigens, induced GCH activity in mouse macrophage-like RAW264.7 cells *(**17**)*. To examine whether GCH expression responds to various pathogens, we stimulated RAW264.7 cells with poly(I:C) as a ligand for TLR3 recognizing double-strand RNA viruses, and R848 as a ligand for TLR7 recognizing single-strand RNA viruses, in place of LPS. Because BH4 is a major form of intracellular biopterins (~90%), we measured total BP after oxidizing BH4 and BH2 to BP instead of measuring labile BH4 alone. We confirmed that addition of 0.1 μg/mL LPS to the medium increased the amount of intracellular BP approximately 8-fold after 24 h compared with non-treated cells (Fig. 1A). Furthermore, addition of 10 μg/mL poly(I:C) to the medium increased the amount of intracellular BP approximately 2-fold after 6 h and 9-fold after 24 h (Fig. 1B), and addition of 0.1 μg/mL R848 increased it approximately 1.5-fold after 6 h and 7-fold after 24 h (Fig. 1C). These data suggest that the intracellular BP levels should be induced by infection with various pathogens not only by bacterial but also by viral origins recognized by corresponding TLRs.

### Induction of GCH transcription and protein expression by immune activation were mediated by a NF-κB pathway

**Fig. 2:**
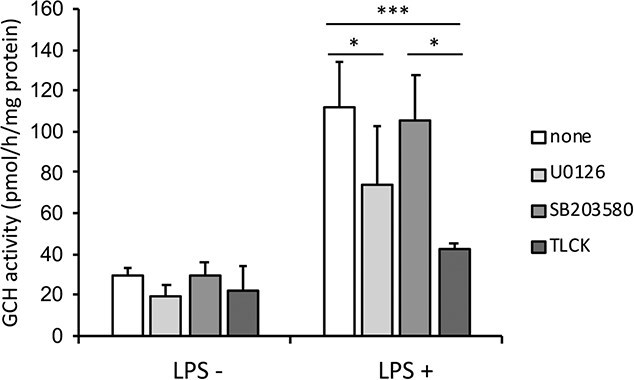
**Suppression of GCH activity upregulated by LPS.** RAW264.7 cells were pretreated with 10 μM U0126, 10 μM SB203580 and 100 μM TLCK for 30 min, and incubated with 1.0 μg/ml LPS for 6 h. The GCH activity was then measured. Data are shown as means ± SD. *n* = 7, 7, 3 and 4 for each treatment. ^*^P < 0.05; ^**^P < 0.01; ^***^P < 0.001; one-way ANOVA with Tukey’s comparison.

To elucidate the mechanism of BH4 production induced by immune activation, we first explored the signaling cascades activated by LPS. We evaluated the effect of various inhibitors for intracellular signals and protein kinases. Cells were pretreated with TLCK, an inhibitor for the NF-κB signal; U0126, an inhibitor for MEK1/2; SB203580, an inhibitor for mitogen-activated protein kinase. We found that TLCK and U0126 significantly suppressed the increases in the GCH activity induced by LPS, while SB203580 did not (Fig. 2). We further analyzed the NF-κB pathway, because it is one of the intracellular signaling pathways downstream of the multiple TLRs, and that is known to be involved in the induction of iNOS for which BH4 acts as a cofactor.

**Fig. 3:**
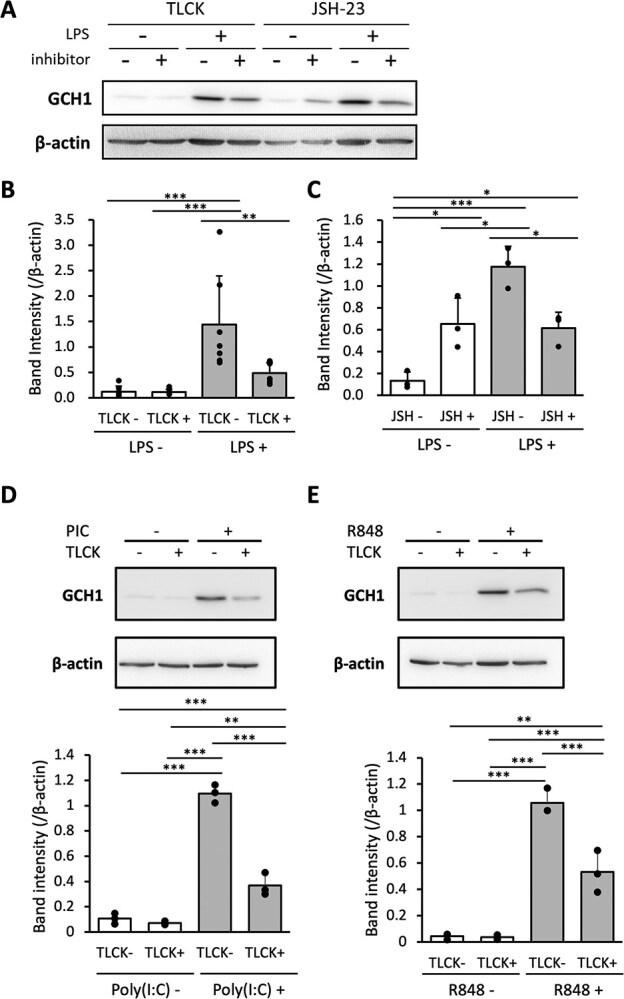
**GCH protein expression upregulated by TLR ligands is suppressed by inhibition of NF-κB pathway. A.** Representative western blot showing whole-cell lysate GCH and β-actin protein levels in RAW264.7 cells that had been prepared with 10 μM TLCK or 30 μM JSH-23 or vehicle for 30 min and then incubated with or without 0.1 μg/ml LPS for 24 h. **B, C.** Ratio of GCH to β-actin intensity of TLCK-treated samples (**B**, *n* = 7) and JSH-23-treated samples (**C**, *n* = 3). **D, E.** RAW264.7 cells were treated with 10 μg/ml poly(I:C) (**D**, *n* = 3) and 0.1 μg/ml R848 (**E,**  *n* = 3), similar to condition **A**.

We performed a western blot analysis to determine the expression levels of GCH proteins in the cells. In this experiment, in addition to TLCK as an inhibitor of the NF-κB pathway, we also used JSH-23, which has a different inhibitory mechanism: i.e. TLCK inhibits IκB degradation *(**22**)*, whereas JSH-23 inhibits nuclear transfer of NF-κB factor RelA (p65) *(**23**)*, to more strongly reinforce the involvement of NF-κB. Intracellular GCH protein levels were upregulated by LPS stimulation, whereas inhibition of the NF-κB pathway by either TLCK or JSH-23 significantly reduced expression levels (Fig. 3A-3C). In addition, TLCK also suppressed the GCH protein levels induced by poly(I:C) and R848 (Fig. 3D and 3E). These results suggest that TLR ligandsinduced GCH expression is mediated by the NF-κB pathway.

**Fig. 4:**
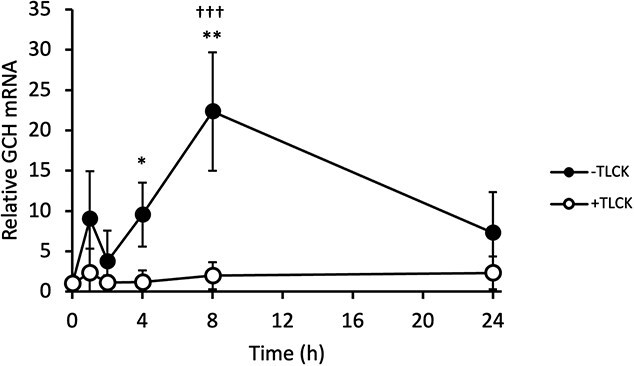
**GCH mRNA expression upregulated by LPS is suppressed by inhibition of NF-κB pathway.** mRNA level of *Gch* was measured in RAW264.7 cells that had been prepared with 10 μM TLCK or vehicle for 30 min and then incubated with 0.1 μg/ml LPS for indicated time. Ratio of *Gch* to *Gapdh* were presented. Data are shown as means ± SD. *n* = 3 for each sample. ^*^P < 0.05; ^**^P < 0.01; Student’s *t*-test for each time point, and ^†††^*P* < 0.001; one-way ANOVA with Dunnett’s comparison for t = 0.

Next, *Gch* mRNA levels were measured at various time points following LPS addition to address the initial response to LPS in RAW264.7 cells. Results showed an increase in mRNA, peaking at 8 h and decreasing by 24 h, although a trend of increase was observed early after LPS addition (Fig. 4). The addition of TLCK almost completely suppressed the increase in mRNA.

**Fig. 5:**
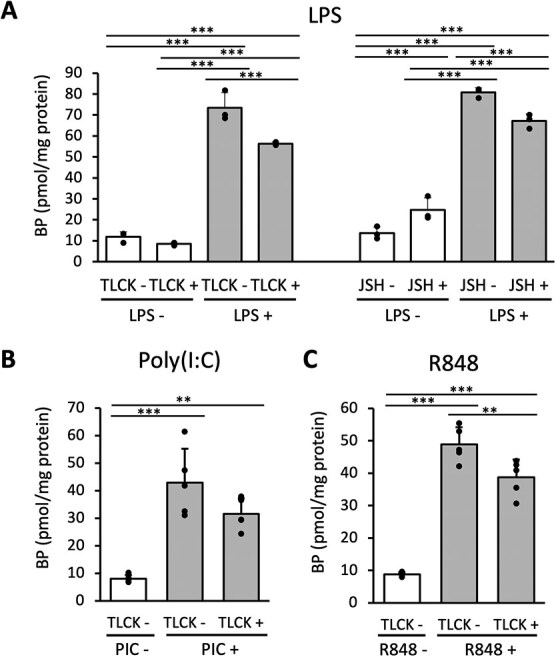
**Intracellular BH4 levels upregulated by LPS is suppressed by inhibition of NF-κB pathway. A-C.** Intracellular BP levels were measured in RAW264.7 cells that had been prepared with 10 μM TLCK or 30 μM JSH-23 or vehicle for 30 min and then incubated with or without 0.1 μg/ml LPS (**A**, *n* = 3) or 10 μg/ml poly(I:C) (**B**, *n* = 5) or 0.1 μg/ml R848 (**C**, *n* = 5). Data are shown as means ± SD. ^*^P < 0.05; ^**^P < 0.01; ^***^P < 0.001; one-way ANOVA with Tukey’s comparison.

To determine whether intracellular BP levels, observed in Fig. 1, were affected by the regulation of GCH via the NF-κB pathway, we performed an analysis using NF-κB pathway inhibitors. We found that intracellular BP levels induced by LPS were significantly suppressed by inhibition of the NF-κB pathway by either TLCK or JSH-23 (Fig. 5A). Furthermore, TLCK showed a tendency to reduce intracellular BP levels induced by poly(I:C) (Fig. 5B), and significantly reduced those induced by R848 (Fig. 5C). These results showed that the regulation of GCH by the NF-κB pathway during immune activation also affected intracellular BH4 level and that the NF-κB pathway was also involved in the regulation of BH4, whereas the suppressive effects of BP levels by these inhibitors seemed to be less than those of GCH protein levels.

**Fig. 6:**
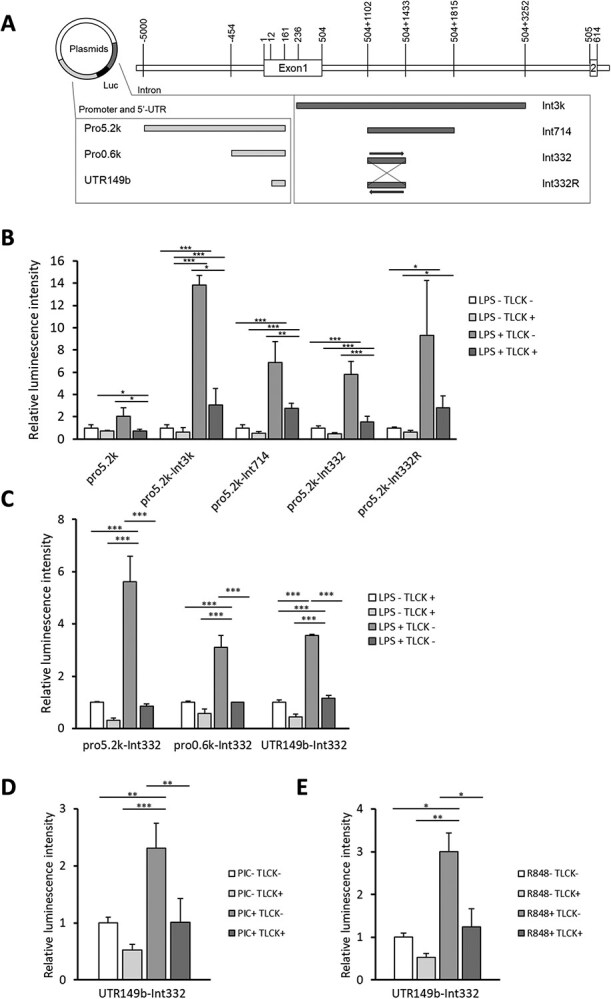
**GCH promoter activity upregulated by TLR ligands is suppressed by inhibition of NF-κB pathway. A.** Schematic of the plasmid vectors used for the reporter assay. The promotor or 5’-UTR of human GCH and the fragments of the indicated regions in intron 1 were inserted into pGV-B2. **B-E.** Relative luciferase activity of each construct was measured in RAW264.7 cells that had been prepared with 10 μM TLCK or vehicle for 30 min and then incubated with or without 0.1 μg/ml LPS (B and D), 10 μg/ml poly(I:C) (D) and 0.1 μg/ml R848 (E) for 24 h. The plasmid vectors used in this experiment are the same as in previous studies *(**17**)* and transfected into cells 24 h before inhibitor treatment. Data are shown as means ± SD. *n* = 3 for each sample. ^*^P < 0.05; ^**^P < 0.01; ^***^P < 0.001; one-way ANOVA with Tukey’s comparison.

Our previous study showed that an enhancer region in intron 1 was required for transcriptional enhancement of GCH by immune activation *(**17**)*. We used the same reporter plasmids as our previous study, which contain the GCH promoter region (pro5.2 k) and regions of various length of intron 1 (pro5.2 k-Int3k, pro5.2 k-Int714, pro5.2 k-Int332, pro5.2 k-Int332R) for the luciferase assay (Fig. 6A). All samples transfected with reporter plasmids containing enhancer regions showed a significant upregulation in GCH reporter activity by stimulation with LPS. The upregulation was suppressed by the inhibition of the NF-κB pathway with TLCK, suggesting involvement of NF-κB pathway in the LPS-induced GCH expression (Fig. 6B).

In addition, reporter assays were further performed with plasmids containing the Int332 and shorter promoter region of 0.5 k or 5’-UTR region of 149b (Fig. 6A). Both constructs, pro0.5 k-Int332 and UTR149b-Int332, showed increased luciferase activity in response to LPS and suppressed with TLCK (Fig. 6C) UTR149b-Int332 also showed increased luciferase activity upon stimulation with Poly(I:C) and R848, which was inhibited by TLCK (Fig. 6D and 6E). These results suggest that both UTR149b and Int332 regions were required for the NF-κB-mediated expression.

### *GCH expression in macrophages* in vivo

**Fig. 7:**
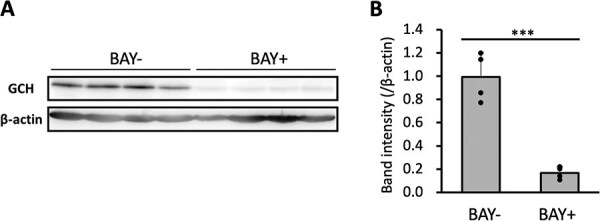
**GCH expression in intraperitoneal macrophages is suppressed by NF-κB pathway inhibition in mice *in vivo*. A**. Western blot showing whole-cell lysate GCH and β-actin protein levels in intraperitoneal macrophages in mice that had been intraperitoneally injected 10 mg/kg BAY11–7082 or vehicle, and 5 mg/kg LPS. LPS was injected 1 h after BAY11–7082 injection, and intraperitoneal macrophages that were collected 6 h later were analyzed. **B**. Ratio of GCH to β-actin intensity. Data are shown as means ± SD. *n* = 4 for each sample. ^***^P < 0.001; Student’s *t*-test.

Finally, we examined whether GCH expression in intraperitoneal macrophages were also regulated by NF-κB as well as RAW264.7 cells. Brewer’s thioglycolate medium was administered intraperitoneally to collect sufficient amounts of macrophages. Four days after the administration, the NF-κB inhibitor BAY11–7082 was administered intraperitoneally, followed by LPS injection 1 h later. Intraperitoneal macrophages were collected 6 h after the BAY11–7082 injection, and the GCH expression levels were analyzed by western blotting (Fig. 7). Although macrophages in non-treated mice exhibited faint bands corresponding to the position of GCH protein (data not shown), we detected strong GCH protein bands in the macrophages of LPS-treated mice. To the contrary, the increased GCH protein bands were almost disappeared in macrophages of mice treated with BAY11–7082. These results indicate that GCH expression in intraperitoneal macrophages were also regulated by NF-κB signals even *in vivo.*

## Discussion

In this study, we demonstrated the involvement of the NF-κB pathway in the induction of GCH expression in immune cells, using RAW264.7 cells and mouse macrophages. The GCH expression upon immune stimulations was suppressed by NF-κB inhibitors. This was observed not only with LPS associated with bacterial infection, but also with TLR ligands relating to detection of RNA viruses (Figs. 1, 3, 5, 6). These results suggested that BH4 plays significant roles in natural immunity to both bacterial and virus infections. Furthermore, the suppression of GCH upregulation upon LPS administration was found in mouse macrophages treated with NF-κB inhibitors (Fig. 7), indicating that NF-κB indeed induces GCH expression in macrophages *in vivo*.

Upon LPS stimulation, not only NF-κB pathway but also MEK1/2 and p38 MAPK pathways are also known to be activated. Although SB203580 as p38 MAPK inhibitor did not suppress the GCH induction, U0126 as MEK1/2 inhibitor treatment significantly suppressed the GCH activity (Fig. 2). We had reported that GCH was induced by NGF through Ras/MEK pathway in dopaminergic PC12D cells *(**14**)*. In the monoaminergic neuron, BH4 is required for the first step of monoamine synthesis catalyzed by tyrosine hydroxylase (TH). TH expression is also regulated by NGF via the MEK1/2 pathway *(**24**)*, suggesting that GCH as a gene for the BH4-synthesis is controlled by the same intracellular signaling pathway as TH, a BH4-requiring enzyme in neuronal cells. While the present result suggests that MEK1/2 pathway is also involved in the induction of GCH activated by LPS in RAW264.7 cells, we focused on the role of NF-kB for induction of GCH by bacterial and viral stimuli in this paper.

In the case of immune cells, BH4 plays a role as a cofactor for iNOS. Nitric oxide (NO) is an important molecule to kill pathogenic bacteria and viruses in the body. Insufficient amounts of BH4 were reported to lead iNOS to be uncoupled, in which an electron transfer required for NO synthesis does not occur, generating superoxide anion instead *(**25**)*. We revealed in this study, that the GCH expression was mainly regulated by the same intracellular signaling pathway as iNOS, as it is well known that expression of the iNOS gene is regulated by the NF-κB pathway. It would underline the importance of BH4 synthesis controlled by the GCH activity for natural immunity.

We evaluated the amounts of BH4 by measuring BP levels in the cells, because it has been known that more than 90% of intracellular BP is present as BH4 *(**1**,*  *5**)* and the strong fluorescence of BP enables us its highly sensitive detection. BH4 is labile compound and easily oxidized to dihydro-forms of BP, *quinonoid*-dihydrobiopterin (*q*BH2) or dihydrobiopterin (BH2) (see Fig. S1). These dihydro-forms of BP are reduced back to BH4 by an enzymatic reaction by QDPR or DHFR. NOS only utilizes BH4 for NO synthesis, and insufficient supply of BH4 results in generation of superoxide anion instead of NO *(**15**,*  *16**)*. Therefore, the control of BH4 synthesis would be critical for immune cells to produce NO or superoxide anion to fight with various pathogens.

We had previously identified the enhancer region required for the induction of GCH by LPS stimulation *(**17**)*. The same region where Ets and C/EBP bind was required for the suppression of upregulation by NF-κB inhibitors, as shown in Fig. 6B. However, the JASPAR database search did not find any NF-κB binding sequences in the enhancer region. This was also the case in the ChIP-Atlas database search in human and mouse. In addition, luciferase activity using the plasmid containing 5’-UTR region is responded to the addition of LPS (Fig. 6C). The JASPAR predicts the binding sequence of NF-κB in the 5’-UTR regions of human *Gch*, and ChIP-Atlas database suggests that NF-κB binds to the promoter and the 5’-UTR regions of the *Gch* gene in human Hodgkin’s lymphoma cell line L1236 and mouse macrophages. The sequence identity of the 5’-UTR regions between human and mouse is 58.5%, also suggesting the potential importance of this region in *Gch* expression (Alignment was shown in Fig. S2). In the case of myosin light chain kinase (MLCK), binding of NF-κB to 5’-UTR region plays an essential role on stimulation-depending expression *(**26**)*. Similarly, NF-κB may regulate *Gch* expression by binding to the 5’-UTR region. Since NF-κB protein interacts with Ets and C/EBP *(**27**,*  *28**)*, NF-κB may bind to the 5’-UTR region and interact with Ets and C/EBP bound to the intron, enhancing GCH expression. However, the binding of NF-κB to the 5’-UTR region of *Gch* gene remains to be clarified.

Inhibition of NF-κB in RAW264.7 cells resulted in a decrease in GCH protein (Fig. 3) and BH4 levels (Fig. 5). However, while GCH was reduced to 34% and 52% for TLCK and JSH-23 treatment compared with LPS alone, intracellular BP was reduced to 77% and 83%, respectively. Although GCH is a rate-limiting enzyme for *de novo* synthesis of BH4, the regeneration pathway is also involved in keeping the BH4 levels in the cell *(**5**)*. It could be the reason for less decreases in the intracellular BP levels compared to the decreases in the GCH protein levels.

In the absence of LPS, treatment with JSH-23 slightly but significantly increased GCH protein and BP levels (Fig. 3C and 5A). The activation of NF-κB is mediated by two pathways, the canonical and non-canonical pathways. The non-canonical pathway is implicated in diverse processes including lymphoid organogenesis, B cell maturation, osteoclast differentiation, and antiviral innate immunity *(**29**)*. Despite the distinct functions of the two pathways, positive (cooperative) and negative crosstalk is reported *(**30**)*. Since proteasomal degradation is involved in the activation via the both pathways, it is likely that TLCK inhibits both pathways. On the other hand, JSH-23, by inhibiting the nuclear transport of RelA (p65), is expected to solely inhibit the canonical pathway, because RelB is utilized in the non-canonical pathway. Therefore, if JSH-23 inhibited only the canonical pathway, the impact of the non-canonical pathway may become more apparent. Kynurenine, which increases during inflammation similar to NP, is synthesized by indoleamine 2,3-dioxygenase as a rate-limiting enzyme. NP and kynurenine were increased by the stimulation with interferon-γ in THP-1 *in vitro*, and the positive correlation between them was observed in patients with COVID19 *(**31**,*  *32**)*. The induction of indoleamine 2,3-dioxygenase was reported to be mediated by the non-canonical NF-κB pathway *(**33**)*. Considering these reports, it is implied that GCH expression may partly regulated by the non-canonical NF-κB pathway.

It has been reported that neuropathic and inflammatory pain was caused by excess amounts of BH4 and inhibitors for sepiapterin reductase, the third enzyme for the *de novo* BH4-synthesis, were effective to reduce pain *(**34**)*. We also reported increases in GCH expression and BH4 levels around the site of incision surgery and the reduction of the postsurgical pain by an inhibitor for sepiapterin reductase *(**35**)*. In addition, administration of a GCH inhibitor promoted polarization of M1 tumor-associated macrophages (TAM), which inhibited tumor growth in syngeneic cancer model *(**36**)*. Vigorous studies for clinical application of NF-κB inhibitors are being conducted against cancers, auto-immune diseases and chronic inflammatory diseases *(**37**,*  *38**)*. A part of these effects of NF-κB inhibitors might be ascribed to suppressive effects of GCH expression.

In conclusion, we revealed that GCH/BH4 is upregulated by NF-κB pathway in macrophages by immune stimulation with TLR ligands for bacterial and viral infection. Considering the induction of iNOS by NF-κB, with BH4 as a cofactor, the GCH upregulation might serve as a mechanism for actively generating NO to defend against these pathogens with natural immunity.

## Supplementary Material

Web_Material_mvaf060
