# Supplementary material for: Transcriptional regulation of the GTP cyclohydrolase I gene via the NF-κB pathway by bacterial and viral immune stimulants
Source: J Biochem. 2025 Oct 23;179(1):51–9. doi: 10.1093/jb/mvaf060 (PMC12779301; doi:10.1093/jb/mvaf060)
Supplement: Web_Material_mvaf060 [file web_material_mvaf060.pdf]

Fig. S1

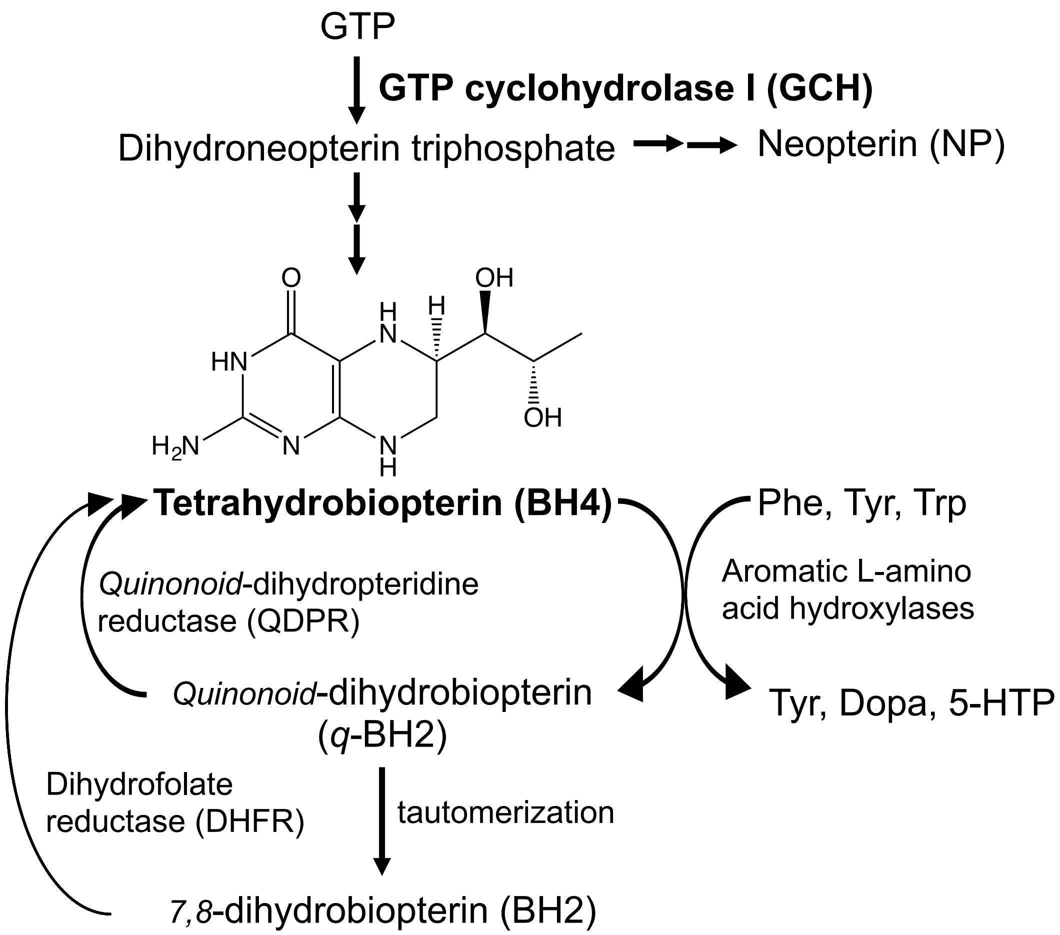

Metabolism of tetrahydrobiopterin (BH4)

Fig. S2

```
human    --CCTGTGGCCGCTCCCGGCTCGGAGTGTGATCTAAGCAGGTTGCGTACCTTCCTCAGGT
mouse    AGCCGGCGACTGCCCTAGACTCCGAGAGTGTCCCTAG-GTGCTGAGC-CCCAGTCCGGGT
           ** * * * ** *  * *** *** ***  *  **   * ** *  **     * ***

human    GACTCCGGCCACAGCCCATTGTCCGCGGCCACCGGCGGAGTTTAGCCGCAGACCTCGA-A
mouse    GAC---GGCCACAGGTTA-------CGGCCGCCGGCTAAGCTGAGCCGCAGCGCTTGTTA
         ***   ********   *       ***** *****  ** * ********  ** *  *

human    GCGCCCCGGGGTCCTTCCCGAACGGCAGCGGCTGCGGCGGGTCC
mouse    GCACCCTAGGGTGTCTC------GGGAGCGGTCGCGCCGGGTCC
         ** ***  ****   **      ** *****  *** *******
```

Alignment of 5’-UTR in human and mouse *Gch* genes. Alignment was generated by using CLUSTALW. Underline indicates predicted NF-kB binding site by JASPAR.
